# Study and Implementation of a High-Quality True Sine Wave DC-to-AC Inverter for Solar Power Generation Systems

**DOI:** 10.3390/mi13101723

**Published:** 2022-10-12

**Authors:** En-Chih Chang, Rong-Ching Wu, Heidi H. Chang, Chun-An Cheng

**Affiliations:** 1 Department of Electrical Engineering, I-Shou University, No.1, Sec. 1, Syuecheng Rd., Dashu District, Kaohsiung City 84001, Taiwan; 2 Department of International Media and Entertainment Management, I-Shou University, No.1, Sec. 1, Syuecheng Rd., Dashu District, Kaohsiung City 84001, Taiwan

**Keywords:** true sine wave DC-to-AC inverter, improved sliding mode reaching law, particle swarm optimization–catfish effect

## Abstract

True sine wave DC-to-AC inverters are becoming more and more important in solar power generation in order to raise the system’s efficiency. A high-quality true sine wave DC-to-AC inverter can be built with a robust intelligent control method. This robust intelligent control method is comprised of improved sliding mode reaching law (ISMRL) and particle swarm optimization (PSO)—catfish effect (CE). The sliding mode reaching law is robust and insensitive to parameter variations and external disturbances. However, it has infinite system-state convergence times and steady-state errors. In addition, solar panels are often affected by partial shading, causing the output power–voltage characteristic curve to be multi-peaked. Such a situation causes misjudgment of the maximum power point tracking with conventional algorithms, which can neither obtain the global extremes nor establish high conversion efficiency. Therefore, this paper proposes an ISMRL based on PSO-CE applied to the tracking of maximum power in the case of partial shading of a solar power generation system. The ISMRL guarantees quick terminable time convergence, making it well-suited for digital implementation. In this paper, PSO-CE is used to find the global best solution of ISMRL, rejecting steady-state errors, slow convergence, and premature trapping in local optimums. Simulation and experimental results are verified using digital implementation based on a Texas Instruments digital signal processor to produce more accurate and better tracking control of true sine wave DC-to-AC inverter-based solar power generation systems.

## 1. Introduction

Following scientific advances, solar power generation is emerging as the fastest long-term investment in terms of cost effectiveness [1,2]. Therefore, how to maximize the performance of solar cells has always been one of the most important development issues all over the world, and it is also the most important problem in solar power generation-related technology [3,4,5]. In order to realize the maximum power tracking of the solar system, it is necessary to adjust the output of the solar cell through the power conversion circuit with the maximum power tracking control function, so that the solar panel can output the maximum power and realize fast and accurate tracking. Various maximum power point tracking (MPPT) methods are proposed throughout the literature, such as bang-bang control, wavelet control, and the Fourier series method [6,7,8,9]. However, the solar illumination and ambient temperature are closely related to the change of the maximum output power of solar panels. Most of these MPPT algorithms cannot strictly analyze the convergence and stability, or cannot quickly track the maximum power point, resulting in the reduction of output power. Sliding mode reaching law (SMRL) is simple and easy to design, and is robust to parameter changes and external disturbances in smooth motion [10,11]; many related SMRL applications have been published in the control of solar power generation systems [12,13,14,15,16,17,18,19,20,21,22,23,24,25,26]. However, in practice, the solar system controlled by the SMRL is affected by uncertainty, the system state convergence time is not limited, and steady-state errors will occur, which will affect the stability, convergence, and performance of the system. Some methods have been proposed to improve steady-state errors, such as predictors and adaptive methodologies. However, they are mathematically complex and computationally time consuming [27,28,29]. Recently, the improved sliding mode reaching law (ISMRL) has provided a terminable system state convergence time to reject the steady-state error (i.e., the terminable time for the system trajectory to reach the sliding mode region in the presence of uncertainty) [30,31,32,33,34,35,36,37,38,39,40,41]. However, it should be noted that even if ISMRL makes the solar power generation system achieve the expected control effect, it is easy for the solar array to become partially shielded by buildings, trees, dust, etc.; this will greatly reduce the output power of the solar system, resulting in a large amount of energy loss. The output power of the solar array will change irregularly and exist in multiple local extremes. If the traditional MPPT methods described earlier are used (such as bang-bang control, wavelet control, and Fourier series method, etc.), they will be tracked to the local extreme value (local maximum power point) rather than the global extreme value (global maximum power point). Several methods have been employed to try to solve multiple local extremums, such as gray wolf optimizer and brute force algorithm [42,43,44]. Although the gray wolf optimizer is fast, it is limited to local searches and cannot conduct global searches. Its disadvantage is that it easily converges to the local solution, while the brute force algorithm shows good solution ability in finding the best solution; that is, it can find a better solution, but its disadvantage is that it needs a long search time and can easily become stagnated on a specific solution. The PSO (particle swarm optimization)-CE (catfish effect) algorithm allows simplified calculation and enhanced population diversity. The PSO-CE algorithm can show the search ability in the global domain and is widely used to solve many optimization problems [45,46,47,48,49,50,51,52,53,54,55,56,57,58,59,60]. It can help address the disadvantage that the traditional PSO algorithm tends to converge to the local extreme value prematurely. Therefore, the PSO-CE algorithm is used to calculate the voltage reference value of the maximum power point of the solar panel under partial occlusion. In this paper, the PSO-CE algorithm is used to search the global extreme of a solar panel under partial occlusion, while the ISMRL is used to track and control it to provide good power output, so that the highest conversion efficiency of the solar system can be maintained. Therefore, the ISMRL based on PSO-CE will improve the steady-state errors, shorten the system state terminable time, solve the multi-peak phenomenon (local maximum power point), and make the solar MPPT system have good steady-state response. The proposed controller is easy to understand, fast converging, easy to program, and able to realize more accurate and stable tracking control. Simulation and experimental results show that the proposed controller will improve the steady-state performance of the solar MPPT system under the conditions of partial shading or high uncertainty. The proposed system is also compared with the traditional SMRL-controlled solar MPPT system to show the superior performance and theoretical applicability of the proposed system.

## 2. Description of Circuit Modeling

**Figure 1:**
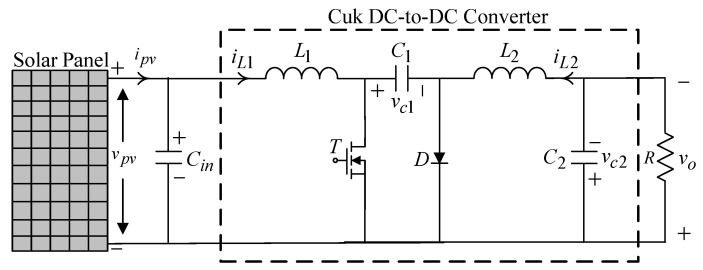
Construction of a Cuk DC-to-DC converter.

A solar power generation system usually consists of a solar panel, a DC-to-DC converter, a true sine wave DC-to-AC inverter, and the attached load. As the illumination and temperature change, there will be a reference value for the voltage corresponding to the maximum power point of the solar power generation system. Thereby, a Cuk DC-to-DC converter (Figure 1) is employed to regulate the solar panel voltage. 

The equation for the dynamics of the Cuk DC-to-DC converter is given by the state space averaging method below:

(1) $$\{\begin{array}{c} {\dot{v}}_{pv}=\frac{i_{pv}-i_{L1}}{C_{in}}\\ {\dot{i}}_{L1}=\frac{v_{pv}}{L_{1}}-\frac{v_{c1}(1-u)}{L_{1}}\\ {\dot{i}}_{L2}=-\frac{v_{c2}}{L_{2}}+\frac{v_{c1}u}{L_{2}}\\ {\dot{v}}_{c1}=\frac{i_{L1}(1-u)}{C_{1}}-\frac{i_{L2}u}{C_{1}}\\ {\dot{v}}_{c2}=\frac{i_{L2}}{C_{2}}-\frac{v_{c2}}{RC_{2}} \end{array}$$

where $\dot{x}={\left[\begin{array}{ccccc} {\dot{v}}_{pv} & {\dot{i}}_{L1} & {\dot{i}}_{L2} & {\dot{v}}_{c1} & {\dot{v}}_{c2} \end{array}\right]}^{T}$, $f(x)={\left[\begin{array}{ccccc} \left(i_{pv}-i_{L1}\right)/C_{in} & \left(v_{pv}-v_{c1}\right)/L_{1} & -v_{c2}/L_{2} & i_{L1}/C_{1} & \left(i_{L2}-(v_{c2}/R)\right)/C_{2} \end{array}\right]}^{T}$, and $h(x)={\left[\begin{array}{ccccc} 0 & v_{c1}/L_{1} & v_{c1}/L_{2} & -(i_{L2}+i_{L1})/C_{1} & 0 \end{array}\right]}^{T}$, and u denotes control input with duty cycle signal.

Then, Equation (1) can be rewritten as

(2) $$\dot{x}(t)=f(x(t))+h(x(t))u$$

**Figure 2:**
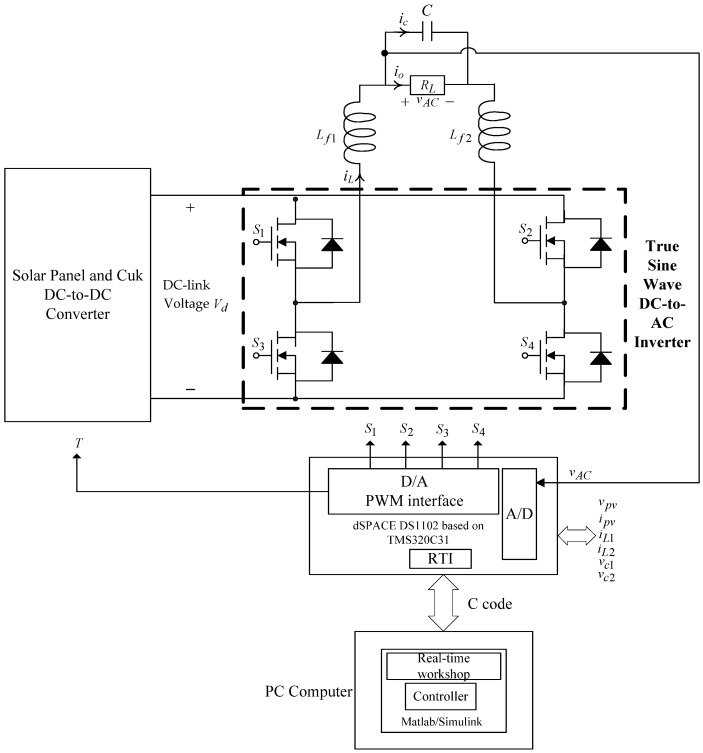
Circuit structure of a true sine wave DC-to-AC inverter.

Let $v_{ref}$ be the voltage reference of the maximum power point calculated by the PSO-CE algorithm, and in order to make $v_{pv}$ follow $v_{ref}$, the ISMRL closed-loop control technology is necessary. In other words, in a PV system, the error in the output voltage can be defined as the state variable $e_{1}=v_{pv}-v_{ref}$. Our goal is to design the control law u properly. If it is well designed, (2) will be stable and the error $e_{1}$ quickly converges to the balance point. The solar output voltage will be the same as the required reference voltage. Even if the solar MPPT system is partially shaded or malfunctioning or under non-matching uncertainty, the tracking control can still be fast, accurate, and robust. Then, a single-phase true sine wave DC-to-AC inverter is used to convert the generated DC power into AC power supplied to the load. A typical true sine wave DC-to-AC inverter is displayed in Figure 2, where four semiconductor switches, LC filters, and loads (resistive loads or capacitive input rectifier loads) are combined. 

The state-space equation for a true sine wave DC-to-AC inverter can be derived by taking the KVL and KCL in Figure 2 as

(3) $${\ddot{v}}_{AC}=-{\dot{v}}_{AC}/R_{L}C-v_{AC}/L_{f}C+K_{PWM}u_{ivt}/L_{f}C$$

where $K_{PWM}$ denotes the equivalent gain of the inverter. 

**Figure 3:**
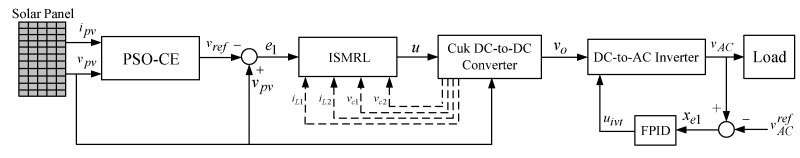
Block diagram of total control system.

The error state equation in an inverter can thereby be described as

(4) $${\dot{x}}_{e2}=-x_{e2}/R_{L}C-x_{e1}/L_{f}C+K_{PWM}u_{ivt}/L_{f}C-v_{AC}^{ref}/L_{f}C-{\dot{v}}_{AC}^{ref}/R_{L}C-{\ddot{v}}_{AC}^{ref}$$

where $L_{f}=L_{f1}+L_{f2}$, $x_{e1}=v_{AC}-v_{AC}^{ref}$, ${\dot{x}}_{e1}=x_{e2}={\dot{v}}_{AC}-{\dot{v}}_{AC}^{ref}$, and $v_{AC}^{ref}$ stands for a demanded sinusoidal reference. Figure 3 plots the structure of the total control system, and in order to allow the error states to converge to zero, the control law $u_{ivt}$ is designed with the fractional proportional–integral-derivative (FPID) method, written as $x_{e1}\cdot (K_{P}+K_{I}\frac{d^{-\gamma }}{dt^{-\gamma }}+K_{D}\frac{d^{\gamma }}{dt^{\gamma }}))$, where $K_{P}$ indicates proportional gain, $K_{I}$ represents integral gain, $K_{D}$ stands for derivative gain, and γ is the fractional order. It is worth noting that at present, for cost and financial considerations, the DC-side input voltage of the true sine wave DC-to-AC inverter obtained from the solar panel and the Cuk DC-to-DC converter in the experimental environment as illustrated in Figure 2 is produced by using a full wave bridge rectifier with a capacitor filter. Under such circumstances, the proposed robust intelligent control method directly controls the inverter to verify the effectiveness. In Figure 3, the proposed robust intelligent control method together with the circuit architecture (including solar panel, converter, and converter) are fully executed with Matlab/Simulink software (R2021a, MathWorks, Natick, MA, USA, 2021) to verify the good performance of the system.

## 3. Control Design

The terminable time sliding function can be written as

(5) $$s=e_{1}+\frac{1}{\varepsilon }e_{2}^{\ell }$$

where $e_{1}=v_{pv}-v_{ref}$, $e_{2}={\dot{e}}_{1}$, ε>0 and 1<ℓ<2. Then, it is advised that the improved sliding mode reaching law be written as follows:

(6) $$\dot{s}=-\eta \left\|e\right\|sign(s)$$

where η>0 and ‖e‖ denotes the norm of state variable. The sign function is replaced by the continuous function $2\pi^{-1}\tan^{-1}(s/\kappa )$ (κ>0) to suppress chatter.

From Equations (2), (5), and (6), the control law is derived as

(7) $$u=-h^{-1}(K_{equ}e+\varepsilon \ell^{-1}e_{2}^{2-\ell })-(2\eta \pi^{-1}\left\|e\right\|\tan^{-1}(s/\kappa ))$$

where $K_{equ}$ signifies equivalent control feedback gain to yield the required sliding mode with system uncertainty at zero. The state of the system is forced to converge to s=0, within a time-terminable period.

**Figure 4:**
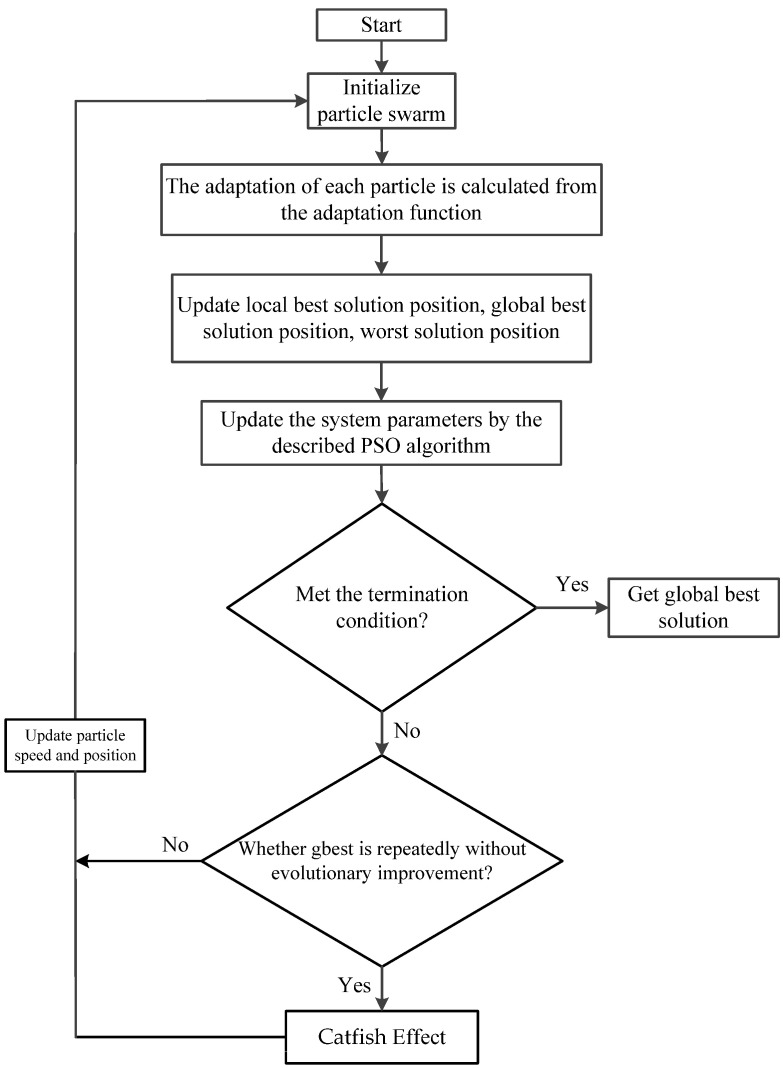
Flow chart of PSO-CE.

Proof: The definition of a Lyapunov candidate is given as follows: $V=s^{2}/2$. In accordance with the dynamical system trajectory along from the control law (7) with the use of the Lyapunov candidate, the time derivative of V becomes

(8) $$\begin{array}{l} \dot{V}=s\dot{s}\\ =s\cdot ({\dot{e}}_{1}+\frac{1}{\varepsilon }\ell e_{2}^{\ell -1}{\dot{e}}_{2})\\ \leq -s\cdot (\frac{1}{\varepsilon }\ell e_{2}^{\ell -1}(2\eta \pi^{-1}\left\|e\right\|\tan^{-1}(s/\kappa )) \end{array}$$

ℓ is fractional (1<ℓ<2), and when $e_{2}\neq 0$, $e_{2}^{\ell -1}>0$; thus, $\dot{V}\leq 0$ holds. Under the situation $e_{2}\neq 0$, it is proven that the system fulfils the Lyapunov stability condition, which allows it to quickly arrive at the sliding surface within a terminable time. To gain the global best solution of the ISMRL control parameters, the PSO-CE algorithm can be used. Each particle represents a potential solution of particle swarm optimization. The goodness of a particle is evaluated by a pre-defined fitness function. In each iteration, the particle searches for the best solution by tracking two extrema (local extrema pbest and global extrema gbest) and flying to a better position in the target search space. Additionally, the mechanism of the worst position is introduced, so that the particles can remember the worst position, and the best path can be determined efficiently during the search process. Thus, the particle updates its velocity and position according to the following equations:

(9) $$\upsilon_{m}^{n+1}=\Omega \upsilon_{m}^{n}+\xi_{1}\kappa_{1}^{n}(X_{pbest,\;m}^{n}-X_{m}^{n})+\xi_{2}\kappa_{2}^{n}(X_{gbest,\;m}^{n}-X_{m}^{n})+\xi_{3}\kappa_{3}^{n}(X_{wt,\;m}^{n}-X_{m}^{n})$$

(10) $$X_{m}^{n+1}=X_{m}^{n}-\upsilon_{m}^{n+1}$$

where $\upsilon_{m}^{n}$ represents present flying speed; $X_{m}^{n}$ represents present position; $X_{pbest,\;m}^{n}$ represents individual best position; $X_{gbest,\;m}^{n}$ represents global best position; $X_{wt,\;m}^{n}$ denotes worst position; $\xi_{1}$, $\xi_{2}$, and $\xi_{3}$ signify learning factors; $\kappa_{1}^{n}$, $\kappa_{2}^{n}$, and $\kappa_{3}^{n}$ are random numbers between 0 and 1; and Ω denotes inertia weight ranging between 0 and 1. The particle swarm optimization algorithm can also be overly concentrated at the local extremes in the early stages of the swarm and can be prematurely reduced, so we introduce the catfish effect into the swarm algorithm to reactivate its global range of search capabilities. The catfish effect is used to stimulate the population dynamics of the sardine population by introducing powerful individuals in the middle of the process to simulate the effect of repelling sardine populations by catfish in nature, thus allowing the population inertia of the fish to change, which can greatly increase the diversity of particles. In other words, the PSO-CE determines that the search swarm has not evolved after several iterations, and that it has fallen into a locally optimal solution, at which point the worst 10% of all particles are removed. A population of (population/10) catfish particles is introduced, which will find the better solution, and these catfish particles guide all the particles to a new region near the best solution. The flow chart of the PSO-CE is displayed in Figure 4.

## 4. Results, Discussion, and Future Research

**Figure 5:**
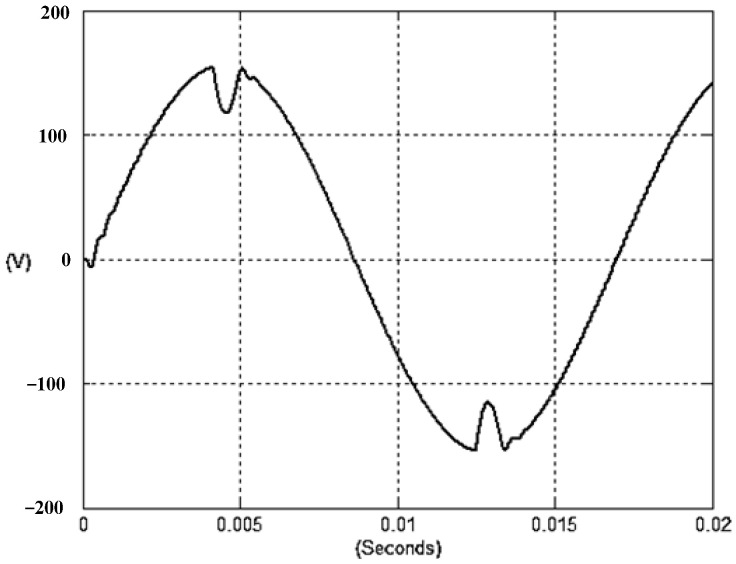
Simulated AC output voltage for the traditional SMRL under a sudden increment of load.

**Figure 6:**
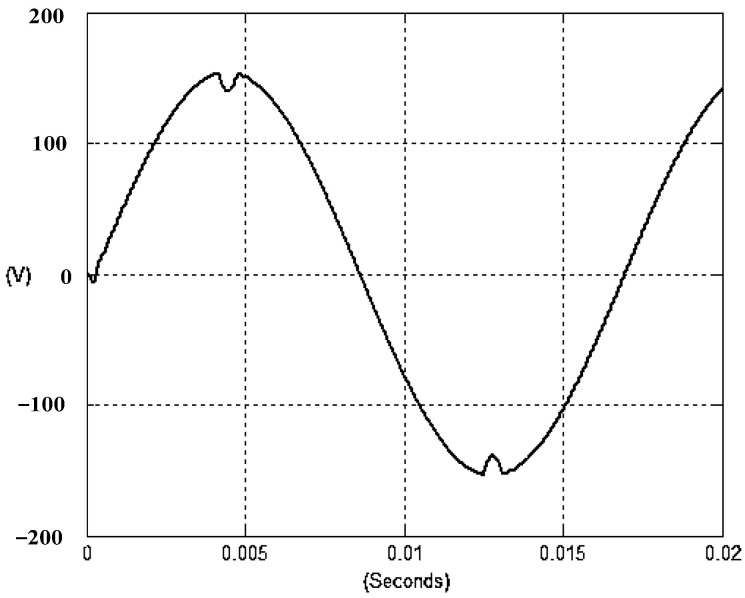
Simulated AC output voltage for the proposed method under a sudden increment of load.

**Figure 7:**
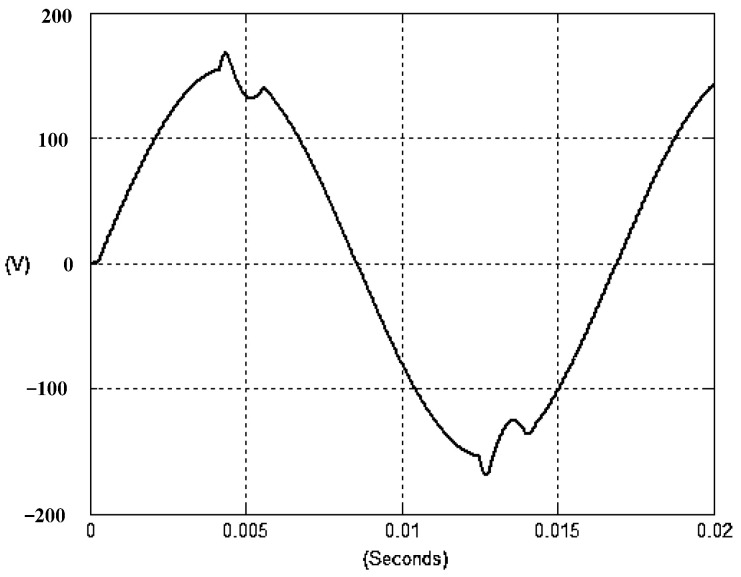
Simulated AC output voltage for the traditional SMRL under a sudden removal of load.

**Figure 8:**
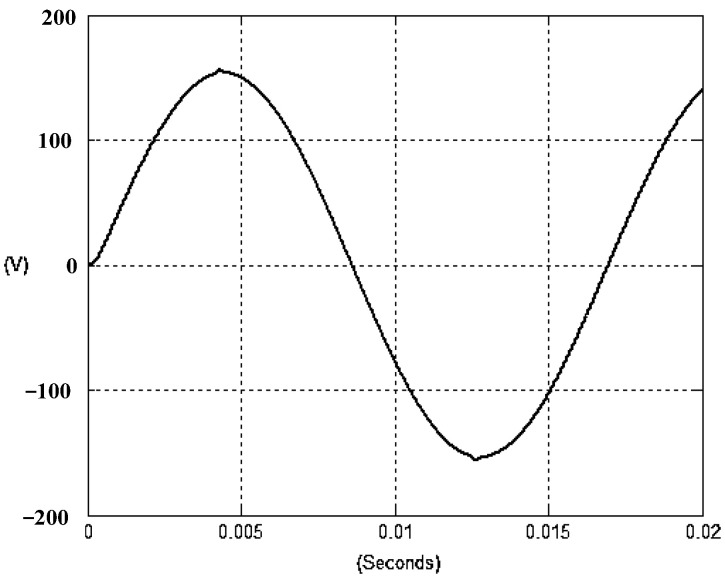
Simulated AC output voltage for the proposed method under a sudden removal of load.

**Figure 9:**
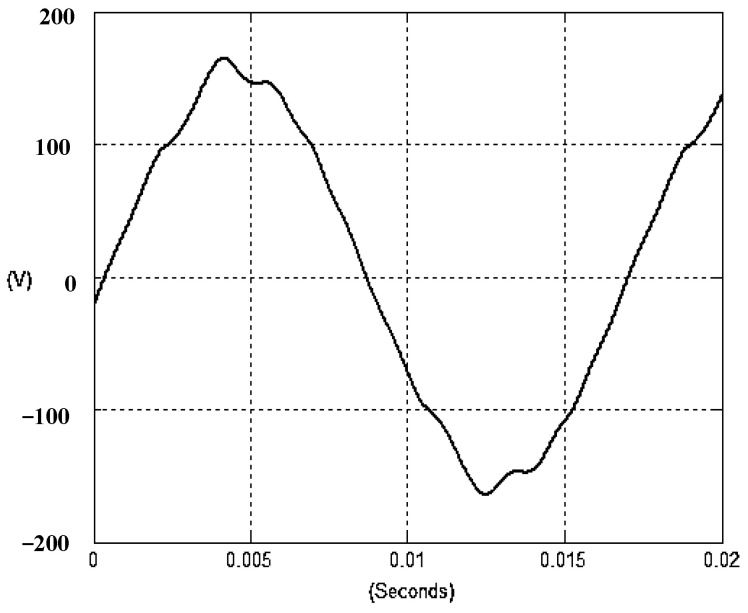
Simulated AC output voltage for the traditional SMRL under rectifier load.

**Figure 10:**
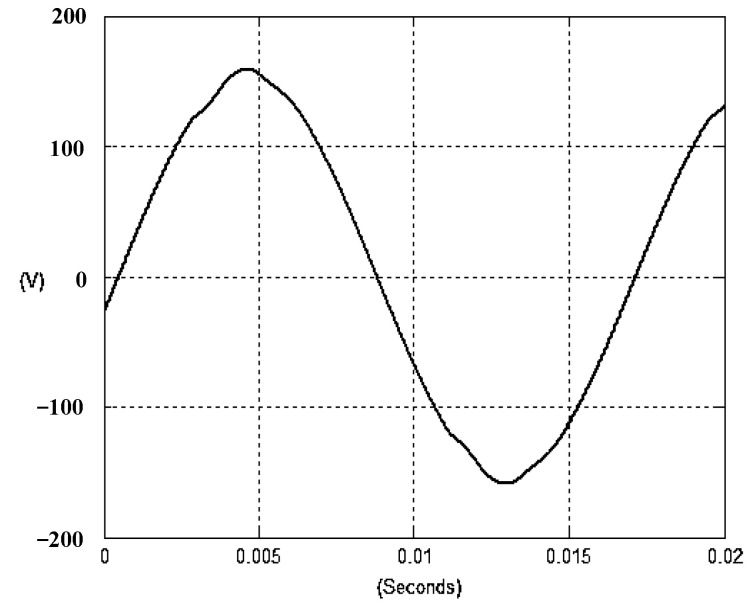
Simulated AC output voltage for the proposed method under rectifier load.

**Figure 11:**
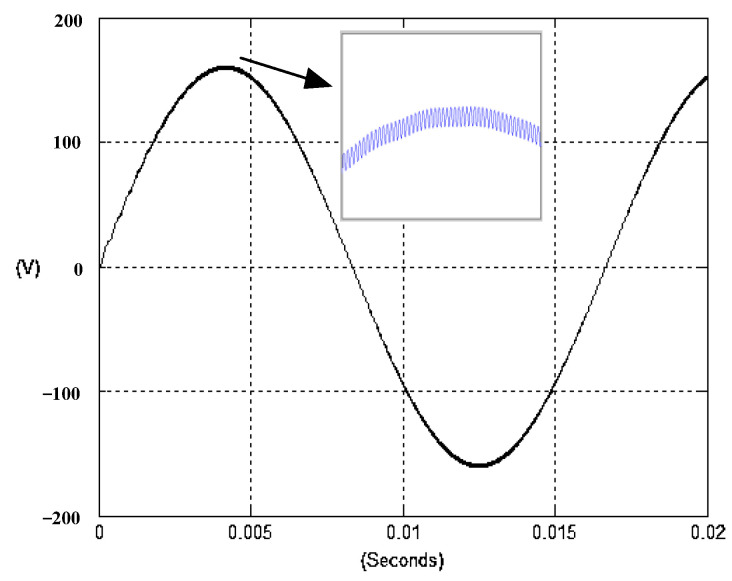
Simulated AC output voltage for the open loop at full load.

**Figure 12:**
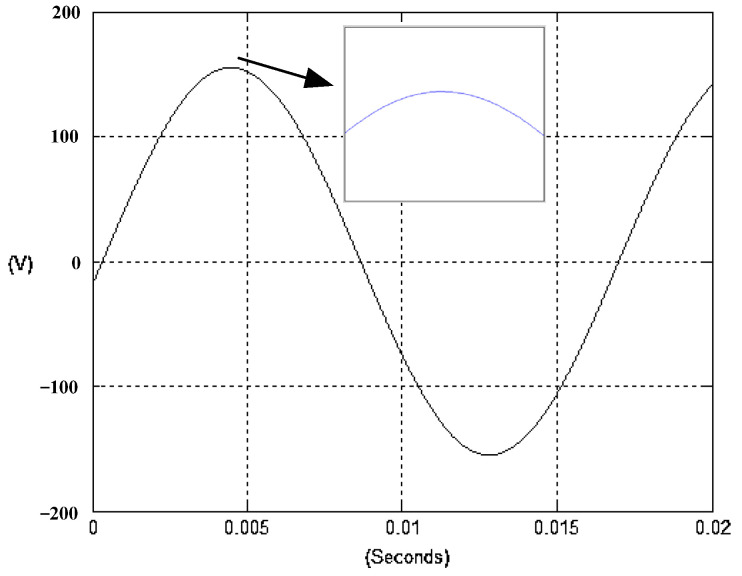
Simulated AC output voltage for the proposed closed-loop method at full load.

**Figure 13:**
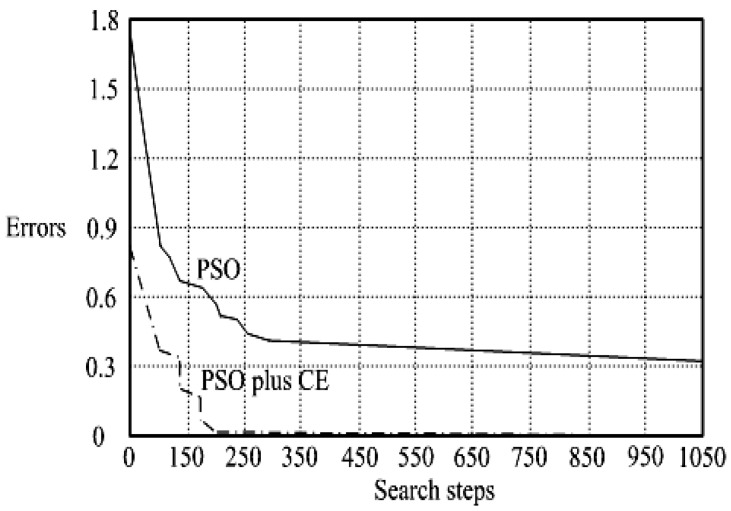
Simulated comparison of PSO-CE and traditional PSO in searching steps under illumination.

**Figure 14:**
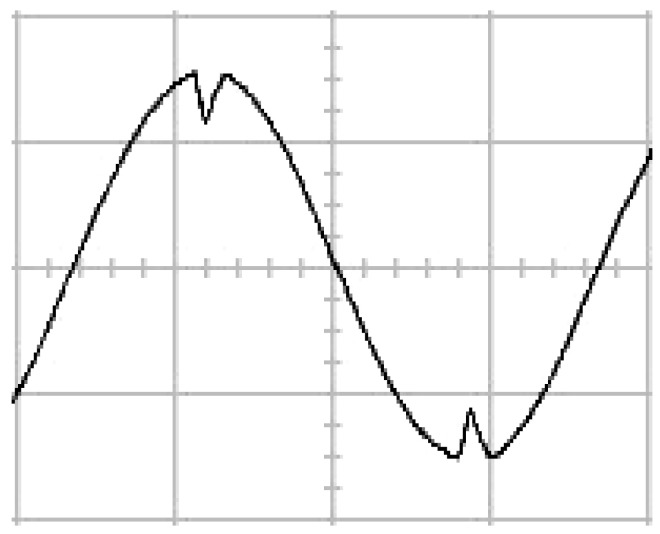
Experimental AC output voltage for the traditional SMRL under a sudden increment of load (vertical: 100 V/div and 5 ms/div).

**Figure 15:**
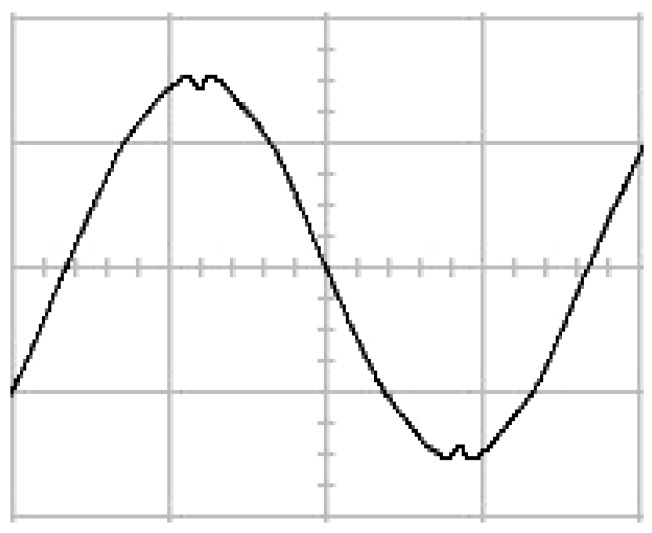
Experimental AC output voltage for the proposed method under a sudden increment of load (vertical: 100 V/div and 5 ms/div).

**Figure 16:**
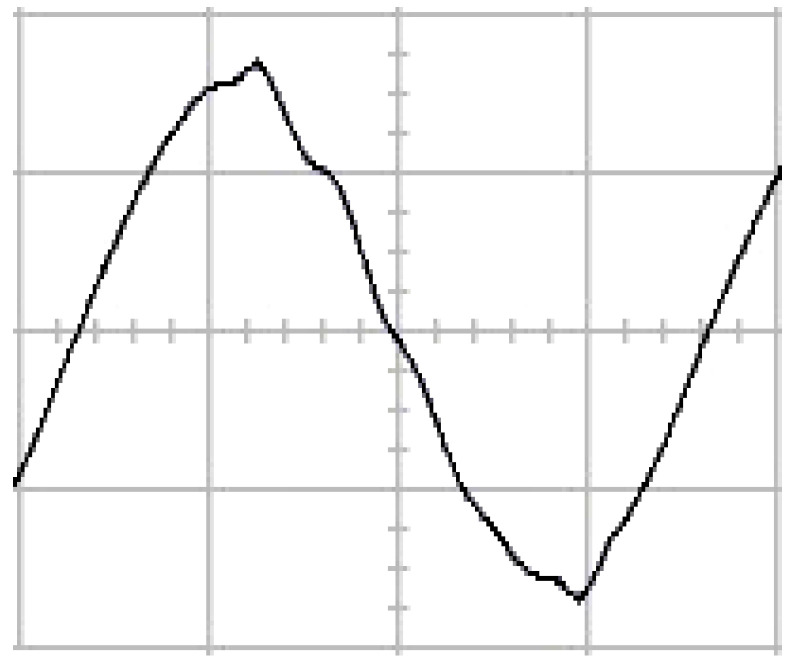
Experimental AC output voltage for the traditional SMRL under a sudden removal of load (vertical: 100 V/div and 5 ms/div).

**Figure 17:**
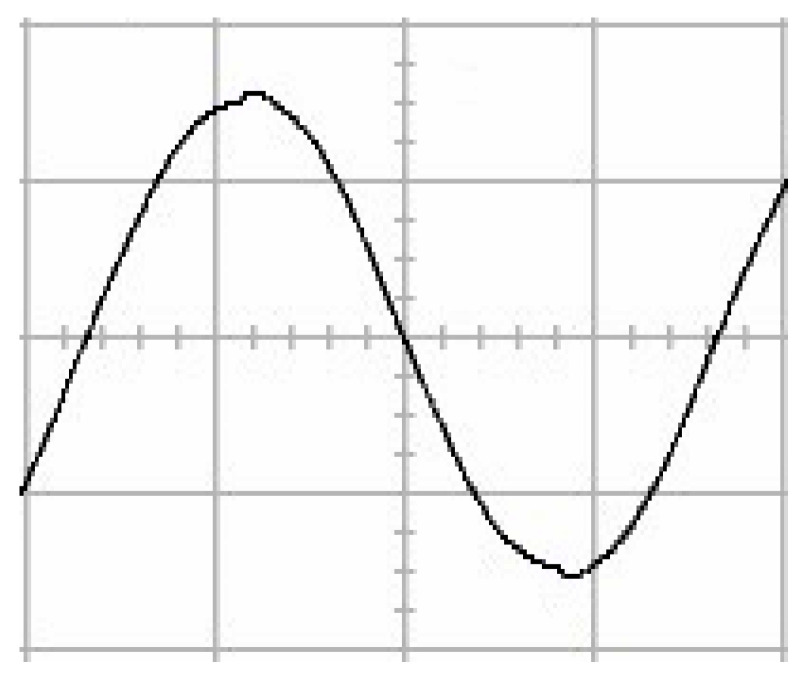
Experimental AC output voltage for the proposed method under a sudden removal of load (vertical: 100 V/div and 5 ms/div).

**Figure 18:**
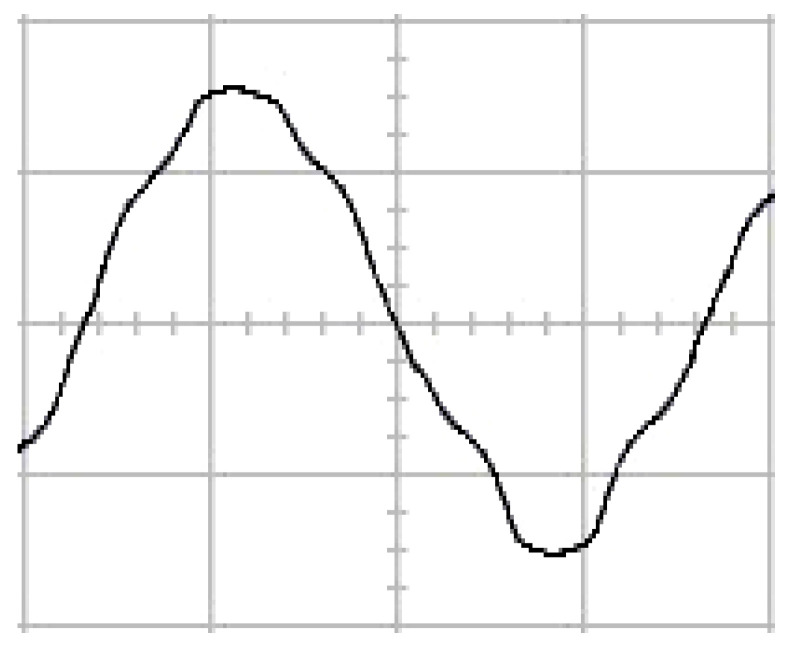
Experimental AC output voltage for the traditional SMRL under rectifier load (vertical: 100 V/div and 5 ms/div).

**Figure 19:**
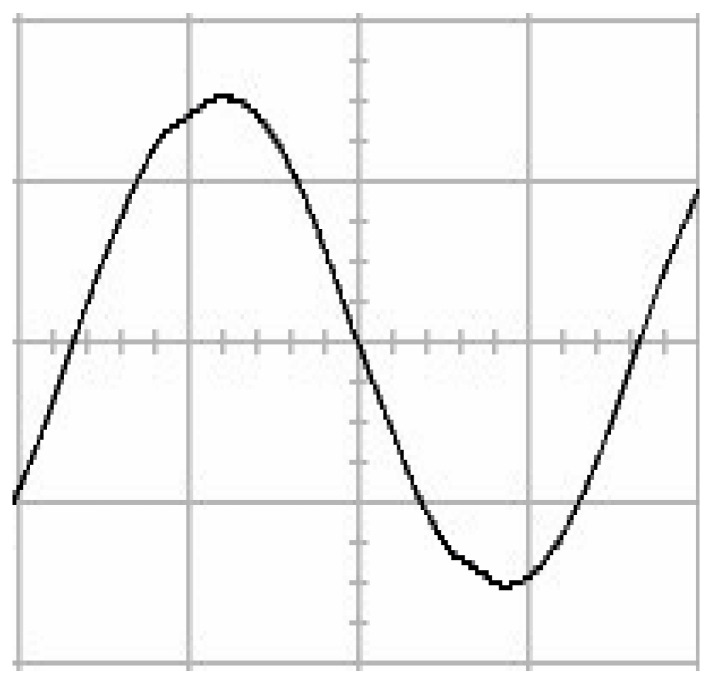
Experimental AC output voltage for the proposed method under rectifier load (vertical: 100 V/div and 5 ms/div).

**Figure 20:**
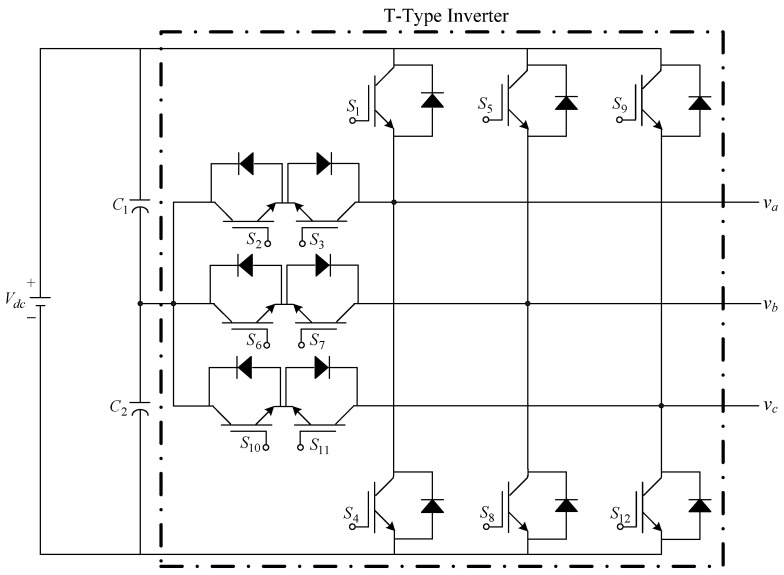
Upcoming research extended to the development of a three-phase T-type inverter.

The presented system parameters are shown in the Table 1. The simulated output voltage waveform resulting from the use of the traditional SMRL (shown in Figure 5) exhibits a substantial drop in voltage along with a slower restoration period during the firing angle. Figure 6 shows the simulated output voltage waveform achieved with the proposed method with a firing angle of 90 degrees per half cycle from no load to full load when loaded with TRIAC. We note that both show good transient behavior and that a small drop in voltage was observed. Following the transient behavior, the voltage waveform comes back to its highly accurate steady state. Operating at TRIAC load with an ignition angle of 90 degrees every half cycle from full load to no load, Figure 7 and Figure 8 show the simulated output voltages using the traditional SMRL and the proposed method, respectively. Checking of the waves revealed that the steady-state reaction in Figure 8 has a rapid response with few fluctuations in comparison to Figure 7. Figure 9 and Figure 10 demonstrate the simulated output voltage waveforms of the true sine wave DC-to-AC inverter by the traditional SMRL and the proposed method when under rectifier load, respectively. The simulated output voltage of the proposed method in the exact steady state appears to be virtually a sine wave, in which it manifests promising AC inverter performance (%THD of 0.05% being obtained in this case); there is a noticeable distortion in the simulated output voltage waveform of the traditional SMRL compared to the proposed method, presenting a high %THD of 25.15%. Figure 11 reveals the simulated inverter output voltage waveform of the open loop at full load, while the simulated output voltage of the closed-loop controlled true sine wave DC-to-AC inverter under full load is displayed in Figure 12. The open-loop control system lacks a feedback signal to judge whether the system output meets the required level, while the closed-loop system allows feedback to govern system states. In the closed-loop compensation system, the output response outperforms the open-loop system, yielding a reduction of the total harmonic distortion. Figure 11 and Figure 12 show that the open-loop system tends to generate oscillating waveforms, causing the system to be unstable. The open-loop system gives great voltage harmonics, which is a major shortcoming with the difficulty in filtering such harmonics. The performance of the proposed method under weak illumination is considered as shown in Figure 13. When compared with traditional PSO, the proposed method has fewer searching steps as well as faster convergence to zero. The experimental output voltage waveform of the traditional SMRL subjected to a sudden increment of load is displayed in Figure 14. Figure 15 depicts the experimental output voltage waveform of the proposed method with a similar loading requirement. The proposed method affords fewer voltage drops and speedier recuperation time at 90 and 270 degrees ignition angles compared to the traditional SMRL. Figure 16 and Figure 17 illustrate the experimental output voltage waveforms with the traditional SMRL and the proposed method, respectively, when the load is suddenly removed at 90 and 270 degrees ignition angles. By examining these waveforms in detail, one can see that there is always a poor steady-state reaction in the output voltage produced by the traditional SMRL controlled solar system, especially in the 90 and 270 degrees ignition angles with a large voltage swing and vibrations. Figure 18 shows the experimental output voltage for the traditional SMRL of the solar system under rectifier load. It can be seen that the output voltage is a distorted sine wave, and the THD value is as high as 26.82%. Figure 19 represents the experimental output waveform of the solar system controlled by the proposed method under rectifier load. The output voltage waveform is very close to the required sinusoidal reference voltage (low %THD value is 0.06%). It can be seen that the proposed solar system has better steady-state response than the traditional SMRL controlled solar system. The comparison of the simulated and experimental THD values for various loads between traditional SMRL and the proposed method is presented separately in Table 2 and Table 3. The results indicate that %THD of the proposed method is low and the voltage waveform is close to the required sinusoidal reference voltage. However, the distortion rate of the output voltage of the solar system controlled by the traditional SMRL is more than 5%, which is worse than the 519 harmonic control standard formulated by the American Society of electrical and electronic engineers. In terms of further research in the future, the true sine wave DC-to-AC inverter architecture in this paper can be extended and developed into a three-phase T-type inverter circuit (as shown in Figure 20) yielding greater power, lower output voltage total harmonic distortion, better efficiency, and reduction of power loss and device stress. The relevant three-phase T-type inverter circuits have been investigated broadly in the latest publications as follows: A fault diagnosis and tolerant control was employed in three-level T-type inverters [61]. The presented control method enables online as well as seamless switching in the configuration of the inverter wiring. The proposed inverter provides a three-phase balancing power output with strengthened robustness. A T-type three-level grid-tied inverter using model-free predictive control was developed [62]. This case removes the influence from the current gradient renewal stoppage whilst also decreasing the calculation complexity, leading to precise inverter output response. A modified T-type topology of a three-phase multi-level inverter with application to photovoltaic systems was presented [63]. The proposed topology features a simplified structure, fewer semi-conductors, and the absence of additional elements. A three-level T-type MLI-based three-phase four-wire distribution static compensator incorporated with a nonlinear sliding mode controller was suggested [64]. This kind of inverter can avoid wave harmonics, reactive power, imbalanced loading, and neutrality current, which are current-dependent power quality issues. Three-phase three-level AC-to-DC (or DC-to-AC) converters with unity power factor operation on the basis of low-frequency partial voltage oscillation were presented [65]. The proposed approach accesses simpler as well as more visual analytic expressions for quantification of partial DC-link voltage vibrations, which allow decreasing low-frequency neutral-point voltage vibrations as well as split DC-link capacitor values.

## 5. Conclusions

Through the proposed method, the PSO-CE algorithm can be used to detect the global maximum power point of the solar array under partial shading. At the same time, the unique benefits of the ISMRL can provide quick convergence of the system state under the conditions of uncertain interference to realize tracking control. The method developed in this way can enable the maximum power output from the solar panel and maintain the highest energy conversion efficiency in the case of partial shading. Under the conditions of sudden load increase, sudden load removal, and rectifier load, the proposed control method shows good steady-state behavior in terms of total harmonic distortion, and the waveform is close to the required sinusoidal reference voltage. However, under the same test load conditions, the output voltage of the traditional SMRL solar system suffers from more than 5% total harmonic distortion. Therefore, the proposed solar power generation system actually produces promising steady-state and transient performance under partial shielding. In final summary, the proposed inverter uses a dSPACE digital signal processor, which has the advantages of fast modeling and suitability for control design. However, the cost of the device is a little higher; in the future, it can be replaced by FPGA (Field Programmable Gate Array) to reduce the overall inverter system cost.

## Figures and Tables

**Table 1:** Parameters of the true sine wave DC-to-AC inverter.

Parameters	Values
Filter inductor, $L_{f}$	0.25 mH
Filter capacitor, C	30 μF
DC link voltage, $V_{d}$	200 V
True sine wave output voltage, $v_{AC}$	110 V_rms_
True sine wave frequency, f	60 Hz
Switching frequency, $f_{sw}$	24 kHz
Load resistance, $R_{L}$	12 Ω

**Table 2:** Simulated AC output voltage total harmonic distortion (THD).

	Methods	Results (%THD)	
**Traditional SMRL**	**Sudden load increase**	**Sudden load removal**	Rectifier load
	THD (%)	THD (%)	THD (%)
	9.83%	9.02%	25.15%
Proposed method	Sudden load increase	Sudden load removal	Rectifier load
	THD (%)	THD (%)	THD (%)
	0.91%	0.56%	0.05%

**Table 3:** Experimental AC output voltage total harmonic distortion (THD).

	Methods	Results (%THD)	
**Traditional SMRL**	**Sudden load increase**	**Sudden load removal**	Rectifier load
	THD (%)	THD (%)	THD (%)
	10.01%	8.89%	26.82%
Proposed method	Sudden load increase	Sudden load removal	Rectifier load
	THD (%)	THD (%)	THD (%)
	1.21%	0.74%	0.06%

## Data Availability

Not applicable.
